# Pyrimidine Azepine Targets the *Plasmodium bc*_1_ Complex and Displays Multistage Antimalarial Activity

**DOI:** 10.1021/jacsau.4c00674

**Published:** 2024-10-07

**Authors:** Juliana Calit, Surendra K. Prajapati, Ernest D. Benavente, Jessica E. Araújo, Bingbing Deng, Kazutoyo Miura, Yasmin Annunciato, Igor M. R. Moura, Miho Usui, Jansen F. Medeiros, Carolina H. Andrade, Sabrina Silva-Mendonça, Anton Simeonov, Richard T. Eastman, Carole A. Long, Maisa da Silva Araujo, Kim C. Williamson, Anna Caroline C. Aguiar, Daniel Y. Bargieri

**Affiliations:** † Department of Parasitology, Institute of Biomedical Sciences, University of São Paulo, São Paulo, SP 05508-000, Brazil; ‡ Department of Microbiology and Immunology, Uniformed Services University of the Health Sciences, Bethesda, Maryland 20814-4712, United States; § Laboratory of Experimental Cardiology, University Medical Center Utrecht, Utrecht University, Utrecht 3584 CS, The Netherlands; ∥ Plataforma de Produção e Infecção de Vetores da Malária−PIVEM, Laboratório de Entomologia, Fundação Oswaldo Cruz-Fiocruz Rondônia, Porto Velho, RO 76812-245, Brazil; ⊥ Programa de Pós-graduação em Biologia Experimental, Universidade Federal de Rondônia/Fiocruz Rondônia, Porto Velho, RO 76812-245, Brazil; # Laboratory of Malaria and Vector Research, National Institute of Allergy and Infectious Diseases, National Institutes of Health, Rockville, Maryland 20852, United States; ∇ Department of Bioscience, Federal University of São Paulo, São Paulo, SP 04021-001, Brazil; ○ Institute of Physics of São Carlos, University of São Paulo, São Carlos, SP 13566-590, Brazil; ◆ LabMol−Laboratory for Molecular Modeling and Drug Design−Faculty of Pharmacy, Federal University of Goias, Goiania, GO 74605-220, Brazil; ¶ Center for Excellence in Artificial Intelligence (CEIA), Institute of Informatics, Universidade Federal de Goiás, Goiania, GO 74605-170, Brazil; & Division of Preclinical Innovation, National Center for Advancing Translational Sciences, National Institutes of Health, Rockville, Maryland 20852, United States; ● Department of Microbiology, Immunology, and Parasitology. Federal University of São Paulo, São Paulo, SP 13563-120, Brazil

**Keywords:** malaria, resistance, pyrimidine azepine, *bc*_1_ complex, *Plasmodium*

## Abstract

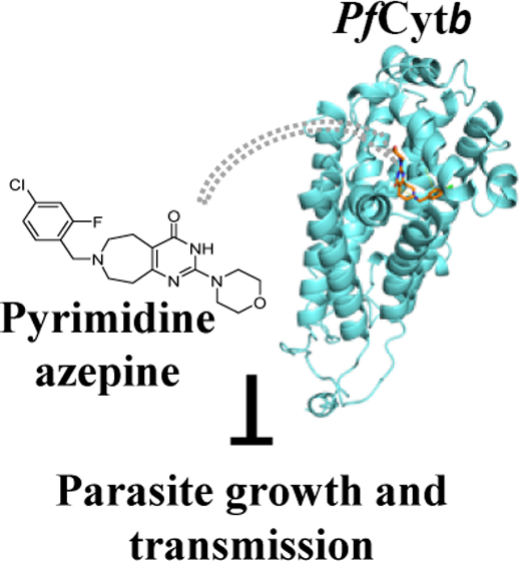

Malaria control and elimination efforts would benefit from the identification and validation of new malaria chemotherapeutics. Recently, a transgenic *Plasmodium berghei* line was used to perform a series of high-throughput in vitro screens for new antimalarials acting against the parasite sexual stages. The screens identified pyrimidine azepine chemotypes with potent activity. Here, we validate the activity of **PyAz90**, the most potent pyrimidine azepine chemotype identified, against *P. falciparum* and *P. vivax* in the asexual and sexual stages. **PyAz90** blocked parasite transmission to the mosquito vector at nanomolar concentrations and inhibited in vitro asexual parasite multiplication with a fast-action profile. Through the generation of *P. falciparum***PyAz90-**resistant parasites and in vitro assays of mitochondrial activity, we identified cytochrome *b* as a molecular target of **PyAz90**. This work characterizes a promising chemotype that can be explored for the future development of new antimalarials targeting the *Plasmodium* cytochrome *bc*_1_ complex.

## Introduction

Malaria is endemic in 85 countries, and in 2022, there were 249 million cases of the disease, resulting in 608,000 deaths. The causative agents of malaria are *Plasmodium* species, transmitted to humans through the bite of infected female *Anopheles* mosquitoes. Two parasite species, *P. falciparum* and *P. vivax*, are responsible for most of the malaria burden worldwide.^1^

Malaria is caused by the multiplication of the parasite’s asexual forms in the human host erythrocytes. The mosquito vectors are infected by circulating nonmultiplicative gametocytes, taken up when they ingest a blood meal from an infected individual. Male and female gametocytes form gametes in the mosquito midgut and fertilize, forming a zygote that matures into the ookinete form.^2^ The ookinete invades the midgut epithelium to the basal lamina and develops into an oocyst,^2^ wherein sporozoites form. The sporozoites migrate to the salivary glands of the mosquito^3^ and are the infectious forms transmitted to humans when inoculated in the skin during the mosquito blood meal, thereby initiating a new infection.

The number of malaria cases and mortality has decreased between 2000 and 2015, primarily due to key interventions such as vector and transmission control and the widespread use of antimalarials. However, the disease numbers have plateaued in the past few years, and resistance to antimalarials is a significant risk for future malaria control efforts.^4^ Thus, developing new antimalarials is critical for the global goal of the elimination of this disease.

In this context, we recently used a mouse parasite *P. berghei* line that expresses a recombinant nanoluciferase (nLuc) reporter only when zygotes are formed, named Ookluc,^5^ to screen thousands of compounds for activity against the parasite sexual stages. Among hundreds of novel compounds identified, pyrimidine azepine chemotypes (Supplemental Figure S1) were potent against *P. berghei* transmission stages and *P. falciparum* asexual stages.^6^ Herein, we investigate the pyrimidine azepine chemotype as a multistage antimalarial, focusing on the transmission-blocking (TB) activity and mode of action against *P. berghei*, *P. falciparum*, and *P. vivax*.

## Results

### Pyrimidine Azepines Block Malaria Transmission

**Figure 1:**
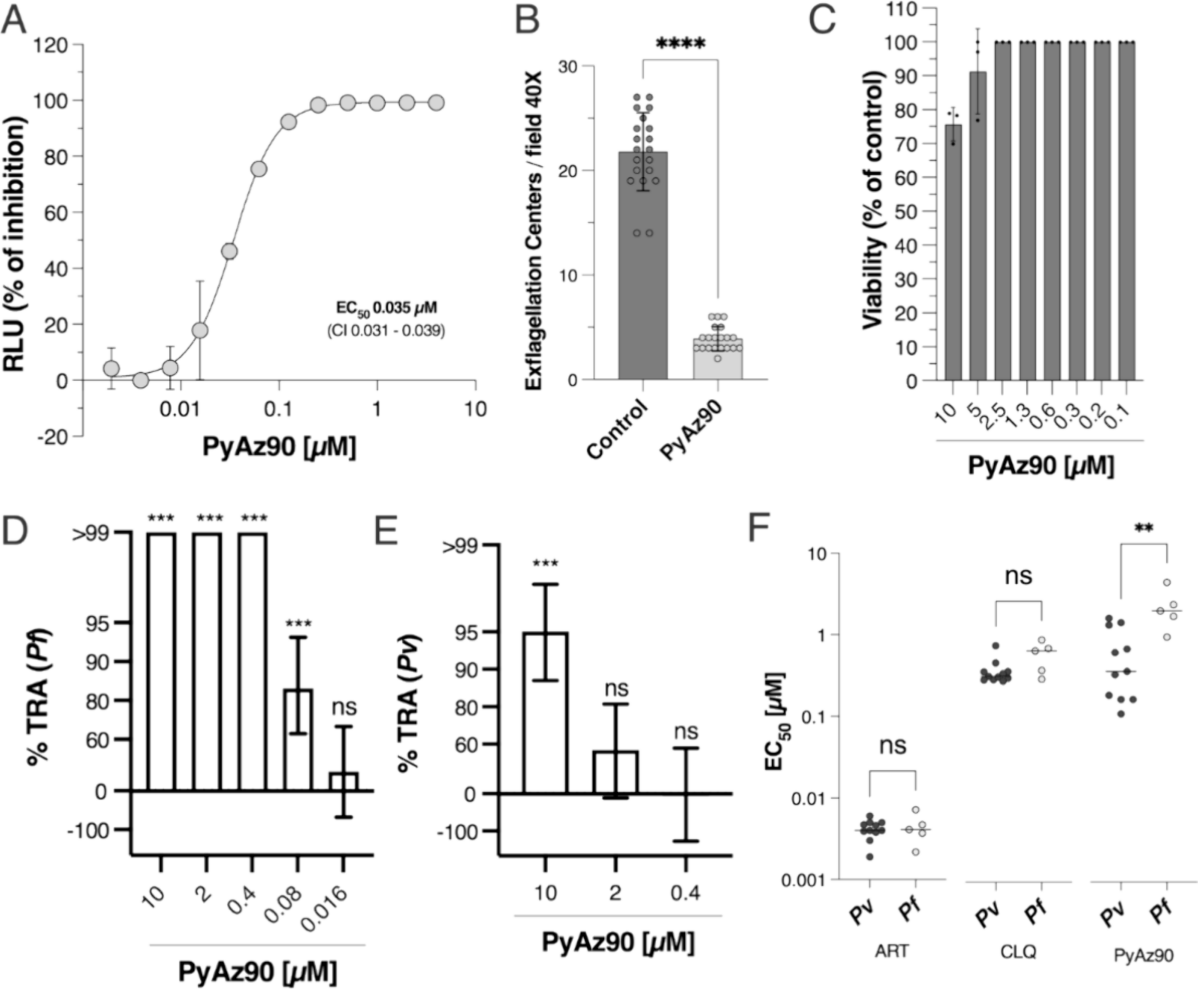
**PyAz90** activity against *Plasmodium* sexual stages. (A) Representative EC_50_ curve of inhibition of *P. berghei* ANKA Ookluc fertilization after treatment with **PyAz90**. The infected blood was subjected to 6 h of incubation at 21 °C with **PyAz90** at different concentrations in triplicates. The percentage of RLUs is the mean + SD of triplicates for each point normalized to the results from the control wells (dimethyl sulfoxide (DMSO) dilutions). (B) Bar graph showing the mean + SD of counted exflagellation centers of *P. berghei* ANKA Ookluc after 14 min of incubation with 10 μM **PyAz90** at 21 °C. Each dot represents the number of exflagellation centers in one individual field. Statistical significance was determined by the Mann–Whitney test. ****p < 0.0001. (C) Effect of different concentrations of **PyAz90** on *P. falciparum* NF54 gametocytes (stages III, IV, and V). Bars represent the mean percentage + SD of viable gametocytes after 48 h of incubation relative to nontreated controls (DMSO dilutions). (D, E) Mean percent inhibition with error bars in oocyst density (%TRA) in mosquito midguts 7 days after *P. falciparum* (D) and *P. vivax* (E) membrane feeding assays in the presence of different **PyAz90** concentrations. Statistical significance and the 95%CI were determined by SMFA-specific^7^ (D) or DMFA-specific^8^ (E) zero-inflated negative binomial models. ***p < 0.001; ns = nonsignificant. (F) EC_50_ against five *P. falciparum* and 11 *P. vivax* field isolates tested using the schizont maturation assay with **PyAz90**, artesunate (ART), and chloroquine (CLQ). Bars represent the median EC_50_ of the drugs for each group of isolates, and the values of the median are also shown. Statistical significance was determined by the Mann–Whitney test. **p = 0.003; ns = nonsignificant.

To explore the antiplasmodial activity of pyrimidine azepines, we selected the most potent compound of this chemotype identified in the previous screen,^6^ the ChemDiv compound S039-3190, which, from now on, we will call **PyAz90**. First, **PyAz90** was tested in vitro for its activity against fertilization using the *P. berghei* Ookluc assay, in which the measured relative light units (RLU) directly correlates with the formation of ookinetes.^5^ The half-maximal drug inhibitory concentration (EC_50_) of **PyAz90** against fertilization was 34 nM (Figure 1A), confirming the previous data (Supplemental Figure S1).^6^ The compound activity against *P. berghei* in vitro fertilization was at least in part due to the inhibition of male gametogenesis, as 10 μM **PyAz90** significantly reduced the number of exflagellation centers formed upon gametocyte activation by up to 80% (Figure 1B). **PyAz90** was also tested against *P. falciparum* NF54 in vitro cultures of gametocytes and was poorly active against these stages, with an EC_50_ greater than 10 μM (Figure 1C). These data suggest that **PyAz90** blocks fertilization not only through cytotoxicity to gametocytes but also by inhibiting gametogenesis. To test whether the in vitro activity of **PyAz90** against sexual stages translates into TB activity, the compound was tested at different concentrations in mosquito membrane feeding assays with *P. falciparum* and *P. vivax*, adding the compound to the infected blood only 2 min before mosquito feeding. **PyAz90** showed 84% inhibition in *P. falciparum* oocyst counts at concentrations as low as 80 nM (Figure 1D and Supplemental Table S1), and *P. vivax* oocyst counts were reduced by 95% with 10 μM of the compound (Figure 1E and Supplemental Table S1), showing that **PyAz90** has TB activity ex vivo against the two most prevalent *Plasmodium* species.

### **PyAz90** Is Active against Both Sensitive and Resistant Parasites

To confirm the activity against asexual stages, **PyAz90** was tested against four *P. falciparum* strains in vitro: the chloroquine-, pyrimethamine-, and mefloquine-resistant Dd2_R539T (MRA-1255), which also bears a single amino acid change in the K13-propeller linked to artemisinin resistance^9^; the parental Dd2 line (MRA-156); and the drug-sensitive lines D6 (MRA-285) and NF54 (MRA-1000). The measured EC_50_ values were 0.96 μM for the Dd2_R539T line, 1.26 μM for the Dd2 line, 0.55 μM for the D6 line, and 0.88 μM for the NF54 line (Supplemental Figure S2). Since the EC_50_ values for all lines were similar and consistently around 1 μM, we chose the Dd2_R539T line for further in vitro experiments because it bears clinically relevant resistance to antimalarials.

Additionally, **PyAz90** was tested against five *P. falciparum* and 11 *P. vivax* field isolates ex vivo in Porto Velho, Rondônia, Brazil, in parallel with the clinically relevant drugs chloroquine and artesunate. All tested isolates were sensitive to **PyAz90**, which was significantly more potent against *P. vivax* than against *P. falciparum* (Figure 1F and Supplemental Figures S3 and S4), with median EC_50_ values of 0.38 and 2.1 μM, respectively, showing that **PyAz90** is active against the asexual stages in the field with a potency comparable to that of chloroquine.

### Identification of the Molecular Target of **PyAz90** in Asexual Stages

To search for the molecular target of **PyAz90**, the reference *P. falciparum* strain Dd2_R539T (MRA-1255), which bears a single nucleotide substitution in the K13-propeller gene leading to an R539T amino acid change conferring more resistance to artemisinin than the parent Dd2 strain, was subjected to high-pressure intermittent selection with 5 times the initial EC_50_ for the selection of a resistant population. After 2 months of intermittent selection cycles, three independent resistant populations from independent flasks were recovered, with EC_50_ shifts 4.5, 3.6, and 4.1 times those of the initial EC_50_ (Supplemental Figure S5). The most resistant population, Dd2_R1, was subsequently retested, in parallel with the parental Dd2_R539T, with **PyAz90** and a panel of antimalarials, and the **PyAz90** EC_50_ shift in the Dd2_R1 was 10.64 times the EC_50_ against the parental strain (Table 1 and Supporting Information, Figure S6). Interestingly, a high EC_50_ shift (20.77 times) was also observed for atovaquone, which specifically targets the *P. falciparumbc*_1_ (*Pfbc*_1_) complex.^10^

**Table 1:** **EC_50_** Values of a Panel of Antimalarials against the Asexual Stages of the PyAz90-Resistant Line^a^

compounds	estimated **EC_50_** [nM] (95% CI Lo, Hi)		
	*P. falciparum* Dd2_R539T	*P. falciparum* Dd2_R1	*shift*
atovaquone	**0.36** (0.31, 0.42)	**7.48** (5.90, 9.60)	20.77
PyAz90	**1210** (1100, 1330)	**12,880** (12,080, 13,810)	10.64
artesunate	**1.86** (1.59, 2.19)	**8.22** (7.61, 8.89)	4.42
amodiaquine	**11.11** (10.78, 11.45)	**5.62** (4.86, 6,52)	0.51
mefloquine	**16.08** (15.21, 17.07)	**7.25** (7.04, 7.48)	0.45
lumefantrine	**2.53** (1.57, 3.54)	**4.98** (4.01, 6.24)	1.97
piperaquine	**30.97** (28.68, 33.15)	**43.12** (40.91, 45.44)	1.39
pyronaridine	**5.27** (4.92, 5.64)	**4.12** (3.81, 4.52)	0.78
ferroquine	**9.62** (9.07, 10.23)	**8.34** (7.33, 9.33)	0.87
chloroquine	**90.05** (83.84, 96.74)	**82.15** (68.66, 100.30)	0.91

a In vitro EC_50_ values (bold) and confidence intervals obtained for a panel of antimalarials against the control *P. falciparum* Dd2_R539T line and the *P. falciparum***PyAz90**-resistant Dd2_R1 line using the SYBR Green method with incubation for 72 h.

**Figure 2:**
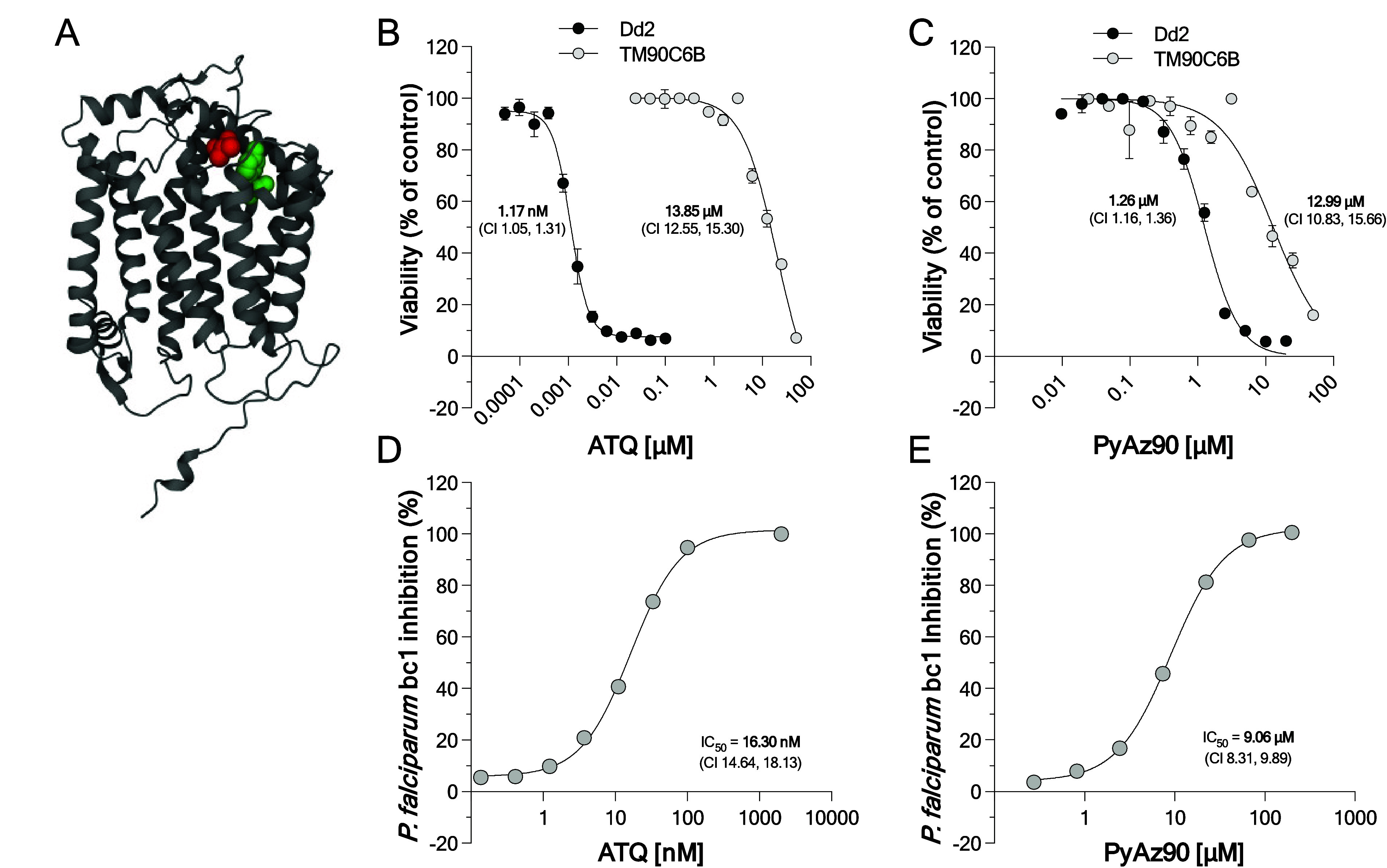
PyAz90 targets the *P. falciparum* cytochrome *b*. (A) 3D structure of the *P. falciparum* cytochrome *b* from Alpha Fold (AF-Q6PPF5-F1). The red residue shows valine 259, substituted by a leucine in all three **PyAz90**-resistant populations generated. The green residue shows tyrosine 268, classically substituted in atovaquone-resistant parasites. (B, C) Concentration–response curves and EC_50_ values of atovaquone (ATQ) (B) and **PyAz90** (C) against the asexual stages of the *P. falciparum* atovaquone-resistant line TM90C6B and the control Dd2 line. EC_50_ values are shown in the graphs. The parasite viability is the mean + SD of triplicates for each point normalized to the results from the control wells (DMSO dilutions). (D, E) Dose–response curves and IC_50_ values of atovaquone (ATQ) (D) and **PyAz90** (E) in vitro against purified *P. falciparumbc*_1_ activity. The percentage of inhibition is normalized to the results from the neutral control wells (DMSO dilutions).

To identify molecular markers of resistance to **PyAz90**, the three generated **PyAz90**-resistant lines, Dd2_R1, Dd2_R2, and Dd2_R3, as well as the parental Dd2_R539T, had their whole genome sequenced using Illumina short read whole-genome sequencing^11,12^ and mapped against the *Pf*Dd2 genome reference using the Burrows-Wheeler Aligner,^13^ and variants were identified using the Genome Analysis Toolkit.^14^ The relevant substitutions were defined by filtering analysis. We considered relevant mutations that were present only in the three generated populations and absent in the control strain (an exhaustive list of mutations can be found in the Supporting Information File 1). Two mutations stood out as lacking in the parental strain and consistently present in all of the three resistant lines: a missense substitution in the PfDd2_100043400 gene, resulting in amino acid change E3698 V, and a missense substitution in the PfDd2_000011300 gene, resulting in amino acid change V259L. The PfDd2_100043400 gene encodes a 9552 amino acid long protein rich in glutamic acid and valine repetitive sequences and is annotated as the gametocyte-specific Pf11-1 protein. PfDd2_000011300 is the mitochondrial gene *cytb*. The same V259L amino acid change was previously reported in an atovaquone-resistant *P. falciparum* NF54 line^16^ and is close to the classic Y268S substitution in *P. falciparum* cytochrome *b* linked to resistance to atovaquone^15^ (Figure 2A). The sequencing results and the 20.77-times shift in the EC_50_ of atovaquone against the **PyAz90**-resistant line Dd2_R1 (Table 1) suggest that cytochrome *b* might be a target of **PyAz90** in *P. falciparum*.

Atovaquone binds to *P. falciparum* cytochrome *b* at the Q_o_ binding site. It acts as a competitive inhibitor of coenzyme Q,^17^ inhibiting the electron flux through the *Pfbc*_1_ complex and collapsing the mitochondrial membrane potential.^18^ The classic Y268S substitution in *P. falciparum* cytochrome *b* likely destabilizes atovaquone binding,^19^ resulting in resistance. Because residues Y268 and V259 are in close proximity (Figure 2A), **PyAz90** was tested against the *P. falciparum* TM90C6B (MRA-205) strain, which has Y268S substitution in cytochrome *b*. The TM90C6B strain is highly resistant to atovaquone, with a > 10,000 times shift in EC_50_ (Figure 2B), and is also resistant to **PyAz90**, although with a more moderate EC_50_ shift of 10.4 times (Figure 2C). Docking analysis of **PyAz90** in a complex with the predicted structure of the wild-type *P. falciparum* cytochrome *b* indicates that it can form π–π interactions with F123 and F264 at distances of 4.9 and 5.3 Å, respectively, which are in close proximity to Y268 and V259 (Supplemental Figure S7) and localized within the conserved binding pocket mapped for atovaquone complexed with cytochrome *bc*1 from *Saccharomyces cerevisiae*.^17^ These results suggest that **PyAz90** and atovaquone interact with similar regions but unique residues in the *P. falciparum* cytochrome *b*.

To confirm that **PyAz90** targets the *Pfbc*_1_ complex, mitochondria were extracted from the *P. falciparum* Dd2_R539T strain and used for in vitro *bc*_1_ activity assays by measuring the cytochrome *c* reduction following the addition of decylubiquinol. The atovaquone control inhibited *Pfbc*_1_ activity in vitro with EC_50_ = 16.3 nM (Figure 2D), and **PyAz90** inhibited *Pfbc*_1_ activity in vitro with EC_50_ = 9.06 μM (Figure 2E), comparable to the activity against asexual parasites. These results are consistent with **PyAz90** directly targeting cytochrome *b* in *P. falciparum*.

### *P. falciparum* Resistance to **PyAz90**

Because the isolation of resistant lines was possible with high-pressure intermittent selection, assays were performed to determine the minimal inoculum of resistance (MIR) of *P. falciparum* Dd2_R539T to **PyAz90**. Cultures were treated with **PyAz90** and DSM265 as the control, an inhibitor of dihydroorotate dehydrogenase (DHODH) known to select resistance in *P. falciparum*.^20^

After 60 days of culture, no resistant parasites were identified in the plate containing an initial inoculum of 1 × 10^7^ parasites and treated with the compound **PyAz90**, demonstrating logMIR > 7. On the other hand, the plate treated with the compound DSM265 showed two resistant parasites identified on days 21 and 32 after the compound was added to the culture, showing an MIR of 4.8 × 10^5^ and a logMIR of 5.7.

### **PyAz90** Antimalarial Activity

**Figure 3:**
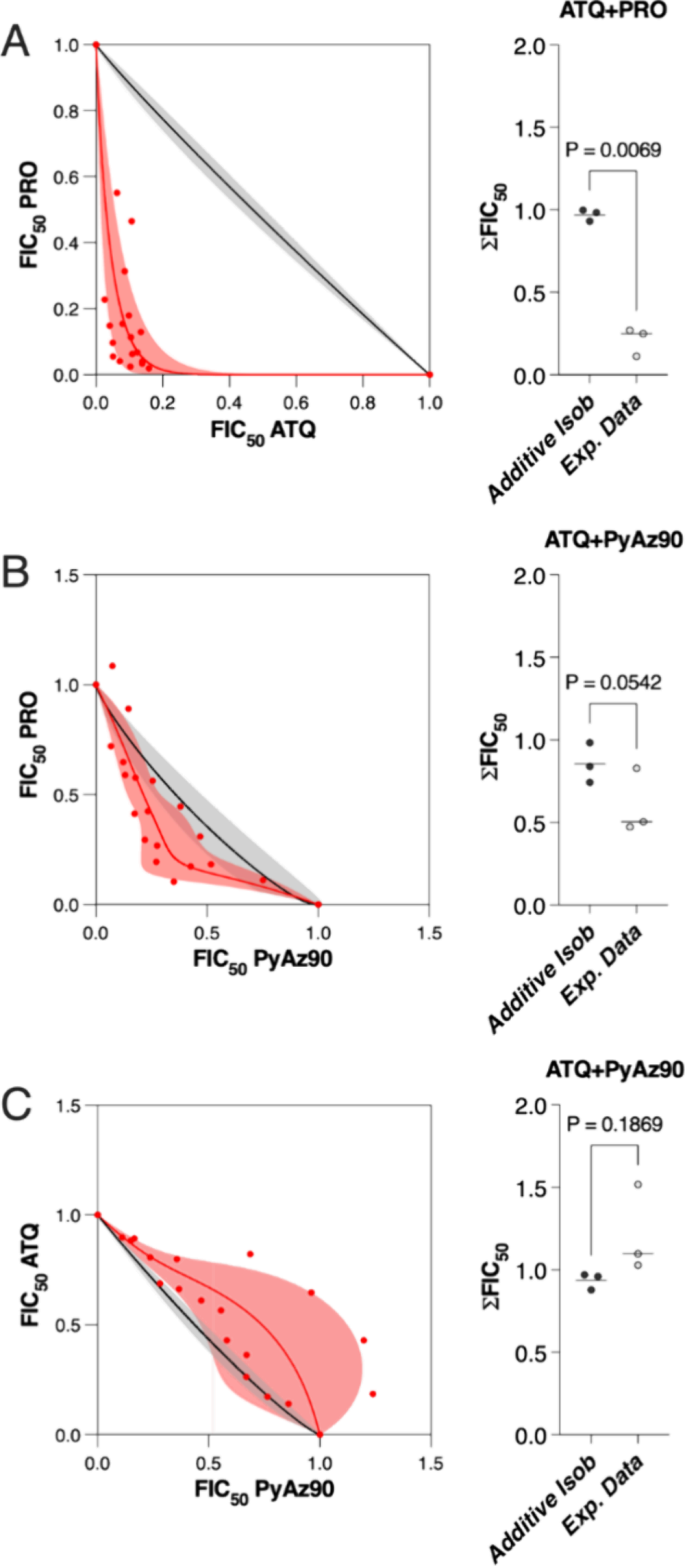
Combined activity of **PyAz90** with atovaquone (ATQ) and proguanil (PRO). Experimental data are represented by red dots, curves, and shaded areas, and the black curves and gray shaded areas represent the modeled data for additive compounds. Isobolograms of atovaquone in association with proguanil (A), **PyAz90** in association with proguanil (B), and atovaquone in association with **PyAz90** (C). The isoboles represent three independent experiments using the *P. falciparum* Dd2_R539T strain. FIC, fractional inhibitory concentration. Graphs on the right of each isobole show the three independent values of ΣFIC_50_ for each drug combination. The *p*-values shown in the graph were calculated by a paired *t* test.

Atovaquone is clinically used as an antimalarial in combination with proguanil, a prodrug that displays synergism with atovaquone by enhancing the latter’s ability to collapse the mitochondrial membrane potential.^21^ To explore whether **PyAz90** also has synergic antimalarial activity with proguanil, mixtures of different concentrations of either drug were tested in vitro against the *P. falciparum* Dd2_R539T strain. As expected, atovaquone and proguanil were synergistic in their antimalarial activity (Figure 3A). Proguanil and **PyAz90** displayed an additive profile, with a trend of synergism (Figure 3B). Considering the three independent experimental data combined, **PyAz90** and atovaquone were additive (Figure 3C). However, in one of the three experiments, the combination showed a profile of antagonism (Figure 3C, right panel).

**Figure 4:**
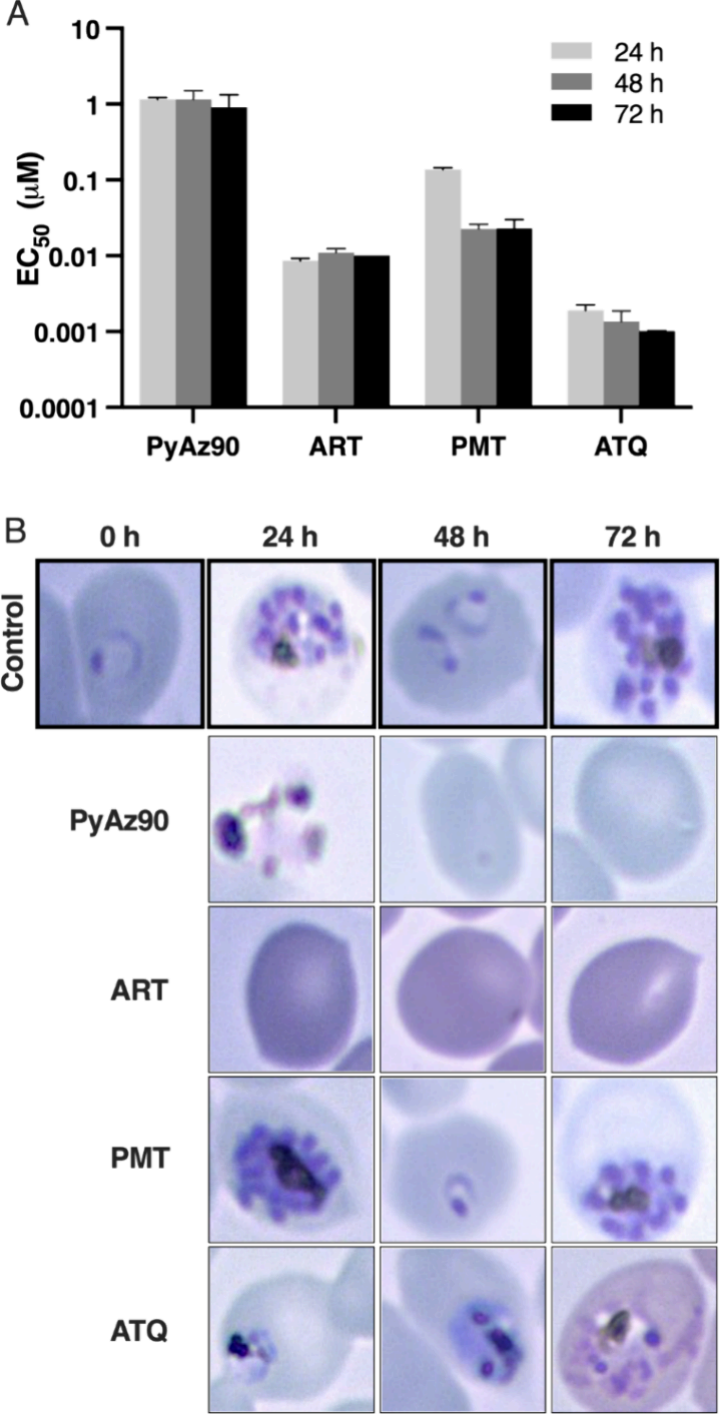
Speed of action and PyAz90 stage target. (A) Mean with SD of the EC_50_ values of **PyAz90**, artesunate (ART), pyrimethamine (PMT), and atovaquone (ATQ) against *P. falciparum* Dd2 asexual stages after 24, 48, or 72 h of incubation. Time 0 h represents the initial morphology after synchronization of the ring-stage parasites. (B) Microscopic images after different times of incubation with each compound.

To test the kinetics of the activity of **PyAz90** against *P. falciparum*, synchronized cultures of the Dd2_R539T strain were treated with **PyAz90**, artesunate, pyrimethamine, and atovaquone for 24, 48, and 72 h, respectively, and the EC_50_ values of each length of treatment was measured at the 72 h time point. The 24 and 48 h assays with artesunate resulted in very similar EC_50_ values compared to the standard 72 h assay. The IC_50_ values of atovaquone and pyrimethamine were higher in the 24 h assays compared to those generated at the 72 h time point. Interestingly, **PyAz90** was a relatively fast-acting drug, as shown by the close EC_50_ values at the earlier time points (Figure 4A) and the absence of parasite forms in the culture in the later time points (Figure 4B). These results indicate that the **PyAz90** activity against *P. falciparum* is faster than the pyrimethamine and atovaquone activities.

## Discussion

In this study, we investigated the antimalarial activity of a pyrimidine azepine compound, **PyAz90**. The compound was effective against *P. berghei* Ookluc fertilization and the asexual stages of *P. vivax* and *P. falciparum* and inhibited mosquito infections by the two most important human species. Prolonged exposure to **PyAz90** led to a resistant *P. falciparum* population with the V259L substitution in cytochrome *b*, within the same region where mutations linked to atovaquone resistance are found. In vitro assays confirmed that **PyAz90** inhibits *P. falciparum* mitochondrial activity, as does atovaquone. However, **PyAz90** activity was additive to proguanil, while atovaquone activity was synergic, and **PyAz90** had a profile of a fast active compound against *P. falciparum* asexual stages.

The multistage activity of **PyAz90** meets several different target product profiles (TPPs) as defined by the Medicines for Malaria Venture (MMV).^22^ Interestingly, **PyAz90** was more active as a TB compound ex vivo than against the asexual stages in vitro. The difference was more pronounced in the case of *P. falciparum*, against which **PyAz90** TB activity is at the nanomolar level. **PyAz90** inhibited *P. berghei* exflagellation, unlike atovaquone, which failed to block exflagellation at 500 μM, as reported previously.^23^ Similarly, **PyAz90** demonstrated activity against ookinete formation with a low EC_50_, unlike atovaquone, which did not inhibit ookinete formation at low concentrations.^23^ Together with the fast-action profile of **PyAz90**, as opposed to the slow-action profile of atovaquone and other exclusive *Pfbc*_1_ inhibitors,^24^ these differences indicate the possibility of an unidentified mechanism of action for **PyAz90**, besides cytochrome *b* inhibition. Another possibility is that the differences above are solely due to **PyAz90** fast inhibition of the mitochondrial electron transport, which in the asexual stages is only essential for pyrimidine biosynthesis^25^ but in male gametocytes is also essential for ATP synthesis.^26^ The **PyAz90** speed of action may be responsible for its ability to inhibit the very fast, energy- and DNA replication-demanding process of male gametogenesis, while the slow atovaquone is not able to act in a similar time frame. Future studies may reveal whether the physicochemical characteristics and kinetics of compound–target interactions support a distinct speed of inhibition of the *bc*_1_ complex by these two compounds.

Also meeting another TPP as defined by the MMV,^22^**PyAz90** was active against multidrug-resistant *P. falciparum* strains, and as recommended by the MMV,^27^ the resistance risk profiling included in this work demonstrates that the generation of resistant mutants had a logMIR > 7 and identifies a resistance molecular marker. Prolonged exposure to **PyAz90** led to a resistant *P. falciparum* population with the V259L mutation in cytochrome *b*. The same mutation was identified by Goodman and colleagues^16^ in atovaquone-resistant strains selected in vitro, which were unable to transmit through mosquitoes. Additionally, the V259L mutation was found when resistant parasites were generated after prolonged exposure to compounds of the thiadiazine class. Interestingly, a significantly reduced susceptibility was observed when parasites containing the V259L mutation were exposed to pyrimidine azepine derivatives,^28^ while drug selection experiments with a pyrimidine azepine compound yielded a G131S mutation in *P. falciparum* cytochrome *b*.

Cytochrome *b* is a validated target in *Plasmodium*, with atovaquone being a *Pf* cytochrome *b* inhibitor used in combination with proguanil in one of the most commonly prescribed medications for malaria prophylaxis. A wider use of atovaquone in antimalarial formulations is hampered by the rapid rise of parasite resistance,^28,29^ which can be at least in part due to the high frequency of mutations in the mitochondrial genome, leading to a high prevalence of resistant parasites in the population. However, atovaquone pharmacokinetics and pharmacodynamics (PK and PD) have also been proposed to influence the rapid selection of atovaquone-resistant parasites.^30^ More specifically, the slow antimalarial activity of atovaquone combined with its high lipophilicity and plasma protein binding levels suggests that, in vivo, atovaquone therapy may subject parasites to low drug concentrations for longer periods, favoring resistance selection. In this context, the speed of action of **PyAz90** may be an advantage of the compound. Moreover, the **PyAz90** predicted LogP of 1.31 is much lower than the atovaquone LogP of 5.1, suggesting different PK and PD in vivo. In addition, while the EC_50_ shifts in **PyAz90**-resistant lines tested were at the order of 10 times the original EC_50_, the shifts for atovaquone EC_50_ in resistant lines are often documented to be 17,000 times.^31^ Together with an MIR higher than that documented for atovaquone,^32^ these data support the consideration of pyrimidine azepine chemotypes as promising scaffolds for the future development of new *Plasmodium* cytochrome *b* inhibitors, with multistage activity and favorable PK, PD, and resistance profiles to thwart atovaquone resistance.

## Materials and Methods

### Antiplasmodial Activity

The activities of compound **PyAz90** (S039-3190, ChemDiv, San Diego, CA, U.S.A.) and known antimalarials (kindly donated by MMV) were assessed against the asexual stages of the *P. falciparum* strains Dd2_R539T (MRA_1255), D6 (MRA-255), NF54 (MRA-1000), Dd2 (MRA-156), and TM90C6B (MRA-205) obtained from MR4 (https://www.beiresources.org/) and the **PyAz90**-resistant Dd2_R1 strain. Parasites were cultured as described previously.^33^ To synchronize the parasites, a 5% D-sorbitol solution (Sigma-Aldrich 240850) was used twice preceding the experiment, with a 10 min incubation at 37 °C each time.^34^ Infected red blood cells (iRBCs) were washed with RPMI medium (Thermo Scientific 23400021) to remove any remaining sorbitol. The compound **PyAz90** was of 93% purity. Analytical analysis was performed on an Agilent LC/MS instrument. A 6.8 min gradient of 4–100% acetonitrile (containing 0.025% trifluoroacetic acid) in water (containing 0.05% trifluoroacetic acid) was used with a 8.5 min run time at a flow rate of 0.8 mL/min. The column was an Agilent Eclipse XDB-C18, 3.5 μm, 3.0 × 75 mm. Purity determination was performed using a diode array detector at 220 nM and an evaporative light scattering detector as backup. Mass determination was performed using an Agilent 6125B mass spectrometer. Data were analyzed using the Agilent Masshunter software.

Parasitemia and parasite morphology were monitored daily using Giemsa stain (Sigma-Aldrich 48900). In vitro experiments were carried out with a culture at 5–10% parasitemia containing primarily ring stages (>80%). The parasite culture was diluted to 0.5% parasitemia and 2% hematocrit in RPMI supplemented with 5% (w/v) Albumax I (Thermo Scientific 11020039) and exposed to various concentrations of the test compounds dissolved in 0.05% DMSO (v/v). Each test was performed in triplicate, and the results were compared with control cultures grown in a drug-free complete medium.

The effects of the compounds were assessed using a SYBR Green assay,^35^ where plates were centrifuged, washed, lysed, and stained with SYBR Green I DNA stain (Thermo Scientific S7585). Fluorescence from uninfected erythrocytes was considered the background, and measurements were performed using a fluorimeter (SpectraMax340PC384) at 485/535 nm. The EC_50_ was determined by curve fitting with software from GraphPad Prism and compared against parasite growth in a drug-free medium.

*P. falciparum* standard membrane feeding assay (SMFA) and *P. vivax* direct membrane feeding assay (DMFA) were performed as described previously.^6^ For *P. vivax*-infected blood, the protocol for blood collection was approved by the Ethics Committee at the Centro de Pesquisa em Medicina Tropical in Rondônia, Brazil (CEPEM-Rondônia), protocol number 28176720.9.0000.0011, and written informed consent was obtained from all volunteers.

### Generating Resistance with Compound **PyAz90** in Dd2_R539T Strains

The high-pressure intermittent selection method described previously was the protocol followed to create a resistant line using *P. falciparum* strain Dd2.^36^ Parasites from Dd2_R539T (MRA-1255), recently cloned, were maintained in three T75 culture flasks (Corning 430720U) under slow shaking to maintain the cell suspension and avoid multiinfected RBCs for 48 h. Then, from these flasks, three T25 culture flasks (Corning 430168U) containing 1 × 10^8^ healthy rings were inoculated with 2.5% hematocrit in 10 mL of culture media, with a high compound pressure, 5× the defined EC_50_ against blood stages (1 μM) for 7 days, with media changes daily, under standard culture conditions. By then, the parasitemia was undetectable by light microscopy, and the compound was removed. The culture medium was changed three times a week, and 1/3 of the cultured blood was changed weekly.

When the parasitemia reached around 2%, after around 60 days, the compound pressure was reinstated twice a week, and the blood change was reduced to preserve the parasites.^36^

When parasitemia stabilized in 5 μM of compound continuously, the parasites were used to assess asexual drug resistance using an SYBR Green assay and gDNA extraction for whole-genome sequencing.

### Whole-Genome Sequencing

The three **PyAz90**-resistant lines—Dd2_R1, Dd2_R2, and Dd2_R3—along with the parental Dd2_R539T strain, had their whole genome sequenced using 150 bp paired end reads using the Illumina Novaseq 6000 device.^11^ An average of 3,266,059 reads per sample were generated and subsequently mapped using the Burrows–Wheeler Aligner against the *Pf*Dd2 reference genome.^13,37^ The average percentage of mapped reads per isolate was 98.5%, and an average 146x coverage was achieved across the genome. The Genome Analysis Toolkit^14^ was then used to identify single-nucleotide polymorphism (SNP) variants per isolate (Supplemental File 1).

The SNP data were analyzed by sorting and selecting the most prevalent mutations in three populations. Genes involved in the asexual cycle of parasites were filtered by relevance based on literature evidence, and duplications were checked in the whole-genome sequencing data, but no relevant duplications were found.

### Assessment of Ex Vivo Activity against Field Isolates from the Brazilian Amazon

This study was conducted in Porto Velho, a city in the Brazilian state of Rondônia, in the Amazon region. All participants provided written informed consent for the ex vivo studies using blood samples before blood collection. Per the national health regulations, all patients received immediate malaria treatment following their participation. The study obtained ethical approval from the Ethics Committee at the Centro de Pesquisa em Medicina Tropical in Rondônia, Brazil (CEPEM-Rondônia), under the registration CAAE 61442416.7.0000.0011.

Patients infected with either *P. falciparum* or *P. vivax* were recruited at the CEPEM. A schizont maturation assay was performed with parasites sourced from patients diagnosed with monoinfections. A total of 26 patients participated, selected based on their lack of antimalarial drug use in recent months and/or presenting malaria symptoms, with parasitemia levels ranging from 2000 to 80,000 parasites/μL.

Exclusion criteria for patient isolates were as follows: (i) initial parasite samples where less than 70% were in the ring stage (*n* = 8); (ii) absence of schizont maturation in test assays (*n* = 2); and (iii) higher counts of inviable parasites than matured schizonts in the untreated controls within the assays (*n* = 1).

For sample collection, 5 mL of peripheral venous blood was drawn into heparin-containing tubes via venipuncture. After removing the plasma and buffy coats, the remaining RBCs were washed and passed through a CF11 cellulose column.^38^ The blood was then diluted to a hematocrit of 2% in either RPMI 1640 medium (for *P. falciparum*)^39^ or IMDM medium (for *P. vivax*) (Thermo Scientific 12200036),^40,41^ each supplemented with 20% compatible human serum.

The parasites were exposed to varying concentrations of the test compound **PyAz90**, ranging from 0.031 to 10 μM, in a hypoxia incubator chamber set to 5% O_2_, 5% CO_2_, and 90% N_2_. The exposure was halted once 40% of the ring-stage parasites evolved into the schizont stage, identifiable by having at least three distinct nuclei in the untreated control wells. The proportion of schizonts per 200 asexual blood-stage parasites was calculated and compared to that of controls. An assay was included in the analysis if the compound was incubated with the parasites for at least 40 h.

### Activities of Compound **PyAz90** and Atovaquone against Cytochrome *bc*_1_

Decylubiquinol for the enzymatic assay was prepared by the reduction of decylubiquinone. A total of 10 μmol of decylubiquinone was dissolved in 400 μL of acidified ethanol (10 mM HCl). A solution of 80 mg of sodium borohydride in 400 μL of distilled water was added to decylubiquinone, and the resulting mixture was shaken to reduce the decylubiquinone. A color change from yellow to colorless caused by decylubiquinol formation was observed after reaction completion. Decylubiquinol was extracted by adding 400 μL of *n*-hexane three times, and the final organic phase was washed with 400 μL of a 2 M NaCl solution in distilled water. The organic phase was once again extracted after phase separation, followed by drying under an N_2_ gas stream in a fume hood. Decylubiquinol was resuspended in 100 μL of acidified ethanol (10 mM HCl), followed by aliquotation and storage at −80 °C. The decylubiquinol concentration was determined spectrophotometrically from absolute spectra, using absorbance (288–320 nm) and an extinction coefficient of 4.14 mM^–1^.^42,43^

*P. falciparum* mitochondria were extracted from the collected parasite of the Dd2_R539T strain. Briefly, iRBCs were lysed with saponin (0.05% m/v solution), followed by several washes with PBS until the supernatant was clear. The parasite was subjected to disruption by nitrogen cavitation (400 psi for 30 min), followed by centrifugation (16,000 g, 4 °C, 1 h). The pellet was resuspended in 2% DDM in 50 mM potassium phosphate buffer, pH 7.4, for 1 h (500 μL of buffer/1000 μL of parasite original pellet). The mitochondrial fraction was obtained after centrifugation (16,000 g, 4 °C, 1 h), and the total amount of protein in the mitochondrial fraction obtained was then quantified using the BCA pierce assay. The assay mixture prepared for the enzymatic assay was composed of 50 mM phosphate buffer (pH 7.4), 2 mM EDTA, 1 mM NaN_3_, 0.03% DDM, and 75 μM cytochrome *c* (C7752, Sigma-Aldrich), and the necessary quantity of purified mitochondrial solution for assays was determined after each extraction by optimization of the signal-to-noise ratios obtained from each batch (200 μg/mL total proteins from the mitochondrial fraction is enough). The reaction was started by the addition of 100 μM decylubiquinol, and cytochrome *bc*1 activity was quantified by indirectly monitoring cytochrome *c* reduction through an increase in absorbance at 550 nm vs 540 nm. The percent inhibition was quantified as a decrease in the slope of the resulting curves in comparison to the positive (2 μM atovaquone) and negative (50 mM potassium phosphate buffer, pH 7.4) controls of inhibition.^42−44^

### Speed of Action Assay

The drug speed of action protocol, adapted from Le Manach et al.,^45^ measures EC_50_ values at three time points. The highest drug concentration on each plate was 10 times the EC_50_ value, with a maximum concentration of 0.5% DMSO in a final volume of 20 μL. Serial 2-fold dilutions of the compound were added to each well containing parasites and incubated for 24, 48, or 72 h under a low-oxygen atmosphere (5% O_2_, 5% CO_2_, 90% N_2_) in a humidified environment at 37 °C.

Postincubation, each RBC pellet was washed 3 times by removing the supernatant and adding 200 μL of fresh culture medium. After this, 200 μL of culture medium supplemented with 5% (w/v) Albumax I was added to each well. The plates were then incubated until 72 h—extending the incubation by 48 h for the 24 h plates and 24 h for the 48 h plates. After this period, the SYBR Green I assay was used to assess parasite viability^35^ and determine the EC_50_ values. Concurrently, a Giemsa-stained smear was performed to evaluate the presence and morphology of the parasites after 24, 48, or 72 h of compound exposure.

Additionally, to assess life stage specificity of the compounds, the morphology of the parasites was examined after exposure to a concentration equivalent to 10 times the EC_50_. Three wells, each containing a synchronized ring-stage parasite suspension at 0.5% parasitemia and 2% hematocrit, were incubated for 24 h under the influence of the inhibitors. The compounds were washed off postinhibition, and incubation was continued until 72 h. The parasite morphology and parasitemia were evaluated at 0, 24, 48, and 72 h using Giemsa-stained blood smears. These treated groups were compared to a control group treated with the vehicle (0.5% DMSO).

### Fertilization and Exflagellation Assays

The analysis of *P. berghei* exflagellation and fertilization followed the protocol outlined by Calit et al.^5^ Two mice were infected with cryopreserved *Pb*Ookluc parasites using intraperitoneal injection. Four days postinfection (dpi), parasitemia and gametocytaemia levels were evaluated by examining blood smears under a light microscope. Blood smears were stained using the “Panóptico Rápido” kit (Laborclin M5B3PZ4ZP), employing a quick hematoxylin–eosin technique to enhance the visibility of the nucleus and cytoplasm. Blood samples with >0.4% gametocytemia were used for further experiments. To maintain optimal gametocyte viability, all materials that came into contact with the mouse’s infected blood were prewarmed to 37 °C during collection via cardiac puncture.

Parasitized blood (2 μL) was diluted in 18 μL of ookinete medium^46^ with serial dilutions of the test compound. Following incubation for 6 h at 21 °C, zygote formation was assessed as a function of luminescence activity measured using the Promega Nano-Glo Luciferase Assay System (Promega N1110), following the manufacturer’s instructions. Light emissions from the samples were quantified by using a plate luminometer at a wavelength of 460 nm.

For the exflagellation assay, 4 μL of fresh mouse blood with at least 0.4% gametocytaemia was mixed with 16 μL of ookinete media in a prewarmed 1.5 mL tube. This mixture was incubated at 21 °C for 12–14 min before being transferred to a microscope slide covered with a cover slide.^47^ Exflagellation centers, where motile male gametes engaged with erythrocytes, were counted in real time under an optical light microscope set to high contrast and low light intensity. Each experiment involved assessing 20 fields under a 40X objective.

### *P. falciparum* Gametocyte Test

Gametocyte isolation was performed as described previously.^48,49^ Initially, an inoculum consisting of 0.2% ring-stage *P. falciparum* NF54 line parasites was prepared in 7% hematocrit (human erythrocytes) and mixed with 12.5 mL of media, comprised of 10.4 g of RPMI (Thermo Scientific 23400021) with 25 mM HEPES, all dissolved in 1 L of sterile water (Thermo Scientific 10977023), and enhanced with 2 g of glucose (Sigma-Aldrich D9434), 2 g of sodium bicarbonate (Sigma-Aldrich S8875), 0.01 g of hypoxanthine (Sigma-Aldrich H9377), 500 μL of gentamicin at a concentration of 50 mg/mL (Sigma-Aldrich G1397), and 10% human serum. The mixture was then added to a T75 culture flask (Corning 430720U, Corning, NY). Following two cycles of daily media changes (without RBC removal), the media volume was doubled, establishing a culture condition of 3.5% hematocrit in 25 mL of media.

After 10 days of daily media changes, 50 mM N-acetyl-d-glucosamine (Sigma-Aldrich A8625) was introduced and maintained for 72 h with continuous daily media changes. By day 13, the majority of the gametocytes were observed to be in stages III–IV.

The Nycodenz (Proteogenix 1002424) gradient method^50−53^ was used for gametocyte purification. The entire content of the culture flask was transferred to a 50 mL conical tube and centrifuged at 800×*g* for 5 min. After the supernatant was carefully removed, 15 mL of warm *P. falciparum* gametocyte growth media was added. Then, 15 mL of a sterile 18% Nycodenz solution in water was gently underlaid, followed by another centrifugation at 800 g for 20 min without brake.

The resulting media/18% Nycodenz interface containing the gametocytes was aspirated with a sterile plastic Pasteur pipet and transferred to 15 mL of warm media. The cells were centrifuged at 800 g for 5 min and washed three times with 15 mL of media.

A MACs column (L.S. Columns—Miltenyi Biotec 130-042-401)^52,54^ pre-equilibrated with 2 mL of warm media was used to remove any remaining uninfected RBCs. 5 mL of concentrated culture was loaded onto each column in a magnetic stand and then washed with an additional 2 mL of warm media. The column was removed from the magnet, and the gametocytes were eluted using 2 mL of warm media.

Gametocyte purity was confirmed via optical light microscopy examination of the Giemsa-stained smears, and the culture flask was then maintained at 37 °C in hypoxic conditions.

To assess drug effectiveness, 5 × 10^4^ gametocytes were added to each well containing media with serial dilutions of the test compounds. Plates were incubated under standard temperature and atmospheric conditions for 48 h. The viability was quantified using the Promega BacTiter-Glo Microbial Cell Viability Assay (Promega G8230), following the manufacturer’s instructions. Luminescence was measured by using a plate luminometer.

### Synergy Assay

The synergy assay was based on SYBR Green fluorescence, utilizing conditions described in the Antiplasmodial Activity section but using two compounds per well, mixed in varying proportions^55^ The tested combinations included atovaquone paired with proguanil (the positive control), atovaquone with **PyAz90**, and proguanil with **PyAz90**, using *P. falciparum* Dd2_R539T strain. The mixture ratios for these combinations were predetermined, ranging from 7:0 to 0:7 across rows A–H of a 96-well plate. A 2-fold serial dilution was performed across the plate, extending horizontally to column 11. Column 12 was designated as a control, containing only RBCs and untreated parasites with four wells allocated for each setup. The stock solution for the compounds, specifically at point 7 following the ratios stated above, was prepared at 32 times the EC_50_ concentration. After 72 h of incubation, the fluorescence levels were measured using the SYBR Green assay as described previously.

Additivity was assessed using the Hand model^56^ with the fractional inhibitory concentration (FIC_50_) values calculated for seven different compound proportions expressed in terms of IC_50_ equivalents. FIC_50_ values from three independent experiments were subjected to nonlinear fitting and statistically compared to the additivity isobole. Absence of a statistical difference between the model and the additivity isobole indicated an additive drug combination, while distinct curves indicated synergy (model below the additivity curve) or antagonism (model above the additivity curve).

### Minimal Inoculum of Resistance

To evaluate the minimum inoculum required to obtain resistant parasites, the Dd2 strain of *P. falciparum* was seeded in a 96-well plate at a density of 1 × 10^5^ parasites per well, suspended in RPMI 1640 medium supplemented with Albumax I at 2% hematocrit. Compound **PyAz90** was added at a concentration that exceeded the previously determined EC_90_ value 3-fold (obtained from the SYBR Green assay with the Dd2 strain). As a positive control for resistant parasite selection, we incorporated DSM265, a known inhibitor of *P. falciparum* DHODH.

During the first week, daily additions of compound **PyAz90** were performed until complete parasite death was confirmed by optical microscopy. Each week, the culture plate was monitored for the emergence of recrudescent parasites by using the SYBR Green method. Additionally, medium changes and compound and RBC replenishment were conducted thrice weekly. The experiment continued for 60 days, allowing assessment of recrudescence and calculation of the minimum inhibitory concentration associated with resistance development.^32^

## Data Availability

Data are available in the main text and supplemental figures and files. Sequencing raw data available upon request.

## Supporting Information Available

Sequencing filtered analysis (XLSX)
SMFA and DMFA data; compound structures; dose–response curves; and molecular docking (PDF)
