# Supplementary material for: Pyrimidine Azepine Targets the Plasmodium bc1 Complex and Displays Multistage Antimalarial Activity
Source: JACS Au. 2024 Oct 7;4(10):3942–52. doi: 10.1021/jacsau.4c00674 (PMC11522906; doi:10.1021/jacsau.4c00674)
Supplement: Supplementary file 2 — au4c00674_si_002.pdf [file au4c00674_si_002.pdf]

Pyrimidine azepine targets the *Plasmodium* bc_1 complex and displays multi-stage antimalarial activity

Juliana Calit[1], Surendra K. Prajapati[2], Ernest D. Benavente[3], Jessica E. Araújo[4,5], Bingbing Deng[6], Kazutoyo Miura[6], Yasmin Annunciato[7], Igor M. R. Moura[8], Miho Usui[2], Jansen F. Medeiros[4,5], Carolina H. Andrade[9,10], Sabrina S. Mendonça[9,10], Anton Simeonov[11], Richard T. Eastman[11], Carole A. Long[6], Maisa da Silva Araujo[4], Kim C. Williamson[2], Anna Caroline C. Aguiar[7,12,*], Daniel Y. Bargieri[1,*]

[1]Department of Parasitology, Institute of Biomedical Sciences, University of São Paulo, São Paulo, SP, Brazil.
[2]Department of Microbiology and Immunology, Uniformed Services University of the Health Sciences, Bethesda, MD, USA.
[3]Laboratory of Experimental Cardiology, University Medical Center Utrecht, Utrecht University, the Netherlands.
[4]Plataforma de Produção e Infecção de Vetores da Malária – PIVEM, Laboratório de Entomologia, Fundação Oswaldo Cruz-Fiocruz Rondônia, Porto Velho, RO, Brasil.
[5]Programa de Pós-graduação em Biologia Experimental – Universidade Federal de Rondônia/Fiocruz Rondônia, Porto Velho, RO, Brasil.
[6]Laboratory of Malaria and Vector Research, National Institute of Allergy and Infectious Diseases, National Institutes of Health, Rockville, MD, USA.
[7]Department of Bioscience. Federal University of São Paulo, São Paulo, SP, Brazil.
[8]Institute of Physics of São Carlos, University of São Paulo, São Carlos SP, Brazil.
[9]LabMol – Laboratory for Molecular Modeling and Drug Design – Faculty of Pharmacy, Federal University of Goias, Goiânia, GO, Brazil
[10]Center for Excellence in Artificial Intelligence (CEIA), Institute of Informatics, Universidade Federal de Goiás, Goiânia, GO, Brasil.
[11]Division of Preclinical Innovation, National Center for Advancing Translational Sciences, National Institutes of Health, Rockville, Maryland, USA
[12]Department of Microbiology, Immunology, and Parasitology. Federal University of São Paulo, São Paulo, SP, Brazil.

[*]Correspondence:
danielbargieri@usp.br
caroline.aguiar@unifesp.br

Table S1. SMFA (*P. falciparum* NF54) and DMFA (*P. vivax* field isolate) data.

SMFA Assay #1

Sample name	Drug conc [μM]	Mean oocyst	Mosquitoes[a]	% inhibition (TRA)[b]			
				estimate	95%CI Lo	95%CI Hi	p-value
Buffer control	0	33.1	37/40				
PyAz90	10	0	0/20	100.0	99.6	100.0	0.001
	2	0	0/20	100.0	99.6	100.0	0.001
	0.4	0	0/20	100.0	99.6	100.0	0.990

SMFA Assay #2

Sample name	Drug conc [μM]	Mean oocyst	Mosquitoes[a]	% inhibition (TRA)[b]			
				estimate	95%CI Lo	95%CI Hi	p-value
Buffer control	0	11.1	29/40				
PyAz90	0.4	0	0/20	100.0	99.6	100.0	0.001
	0.08	1.9	6/20	83.8	63.9	93.5	0.001
	0.016	8.2	8/20	28.8	-59.6	68.1	0.427

DMFA Assay #1

Sample name	Drug conc [μM]	Mean oocyst	Mosquitoes[a]	% inhibition (TRA)[b]			
				estimate	95%CI Lo	95%CI Hi	p-value
Buffer control	0	314.7	40/40				
PyAz90	10	15.7	39/40	95.0	87.7	97.9	0.001
	2	140.7	40/40	55.3	-8.4	81.0	0.075
	0.4	328.6	40/40	-4.4	-141.0	57.2	0.932

[a] Number of infected mosquitoes / Number of dissected mosquitoes

[b] Statistical testing is based on a zero-inflated negative binomial random effects model.

Figure S1. Pyrimidine azepine chemotypes previously identified with activity against *Plasmodium* berghei sexual stages [1]**.** The ID numbers at the NIH National Center for Advancing Translational Sciences, formerly known as the NIH Chemical Genomics Center (NCGC), ID code from ChemDiv (supplier), and the AC_{50}s are shown.

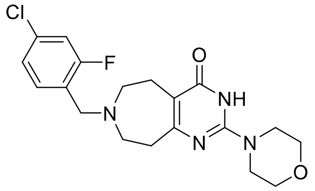

NCGC00440653-01

ChemDiv S039-3190
AC_{50} = 63 nM
PyAz90

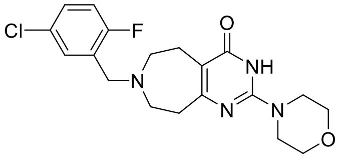

NCGC00440651-01

ChemDiv S039-3182
AC_{50} = 117 nM

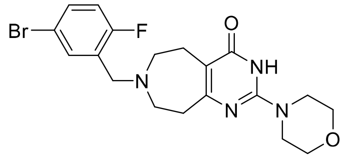

NCGC00440663-01

ChemDiv S039-3202
AC_{50} = 246 nM

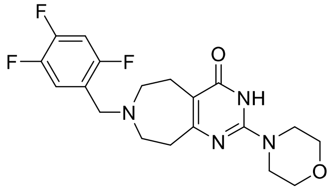

NCGC00440659-01

ChemDiv S039-3197
AC_{50} = 276 nM

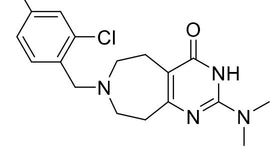

NCGC00440505-01

ChemDiv S039-1700
AC_{50} = 497 nM

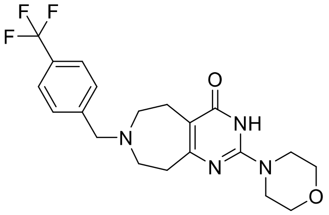

NCGC00440629-01

ChemDiv S039-3153
AC_{50} = 658 nM

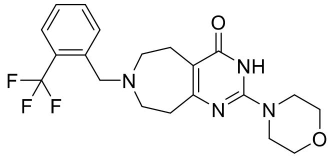

NCGC00440637-01

ChemDiv S039-3166
AC_{50} = 708 nM

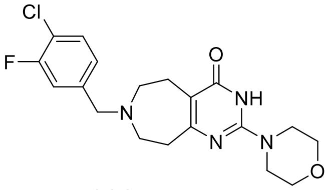

NCGC00440655-01

ChemDiv S039-3191
AC_{50} = 708 nM

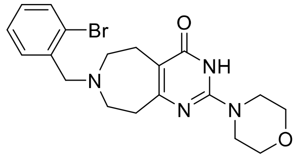

NCGC00440625-01

ChemDiv S039-3146
AC_{50} = 794 nM

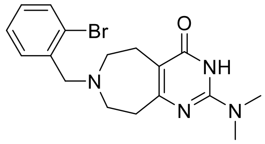

NCGC00440591-01

ChemDiv S039-2589
AC_{50} = 930 nM

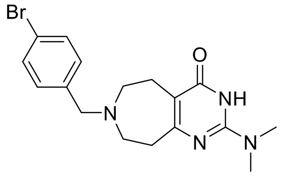

NCGC00440593-01

ChemdDiv S039-2590
AC_{50} = 930 nM

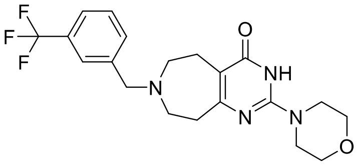

NCGC00440639-01

ChemDiv S039-3167
AC_{50} = 1171 nM

Figure S2: Dose-response curves of PyAz90 against the *P. falciparum* strains Dd2_R539T, D6, NF54 and Dd2. The parasite viability is the mean of duplicates for each point normalized to the results from the control wells (DMSO dilutions). EC_{50} values and 95% Confidence Intervals (CI) are shown.

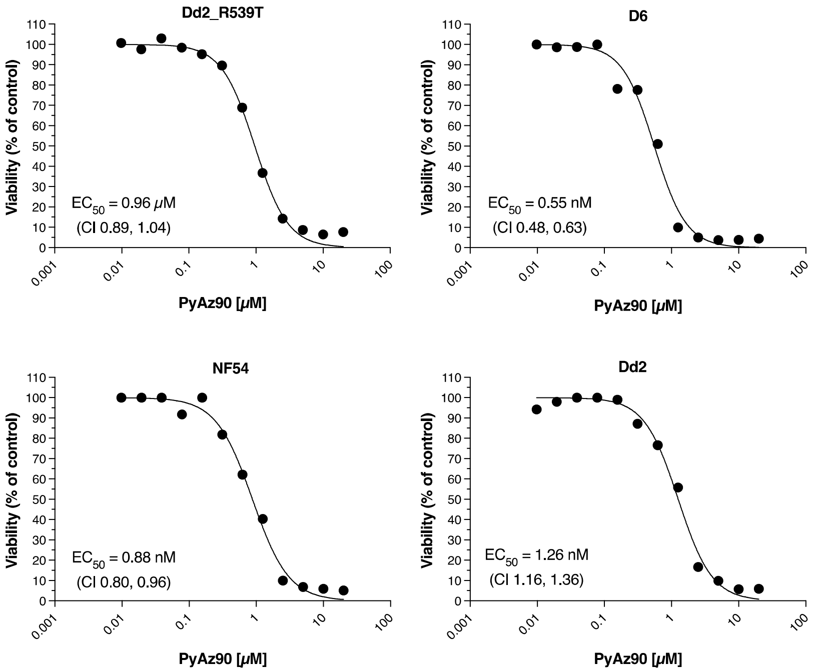

Figure S3: Dose-response curves of PyAz90, chloroquine (CQ) and artesunate (ART) against eleven *P. vivax* (Pv) isolates. The parasite viability is normalized to the results from the control wells (DMSO dilutions). IC_{50} values are shown.

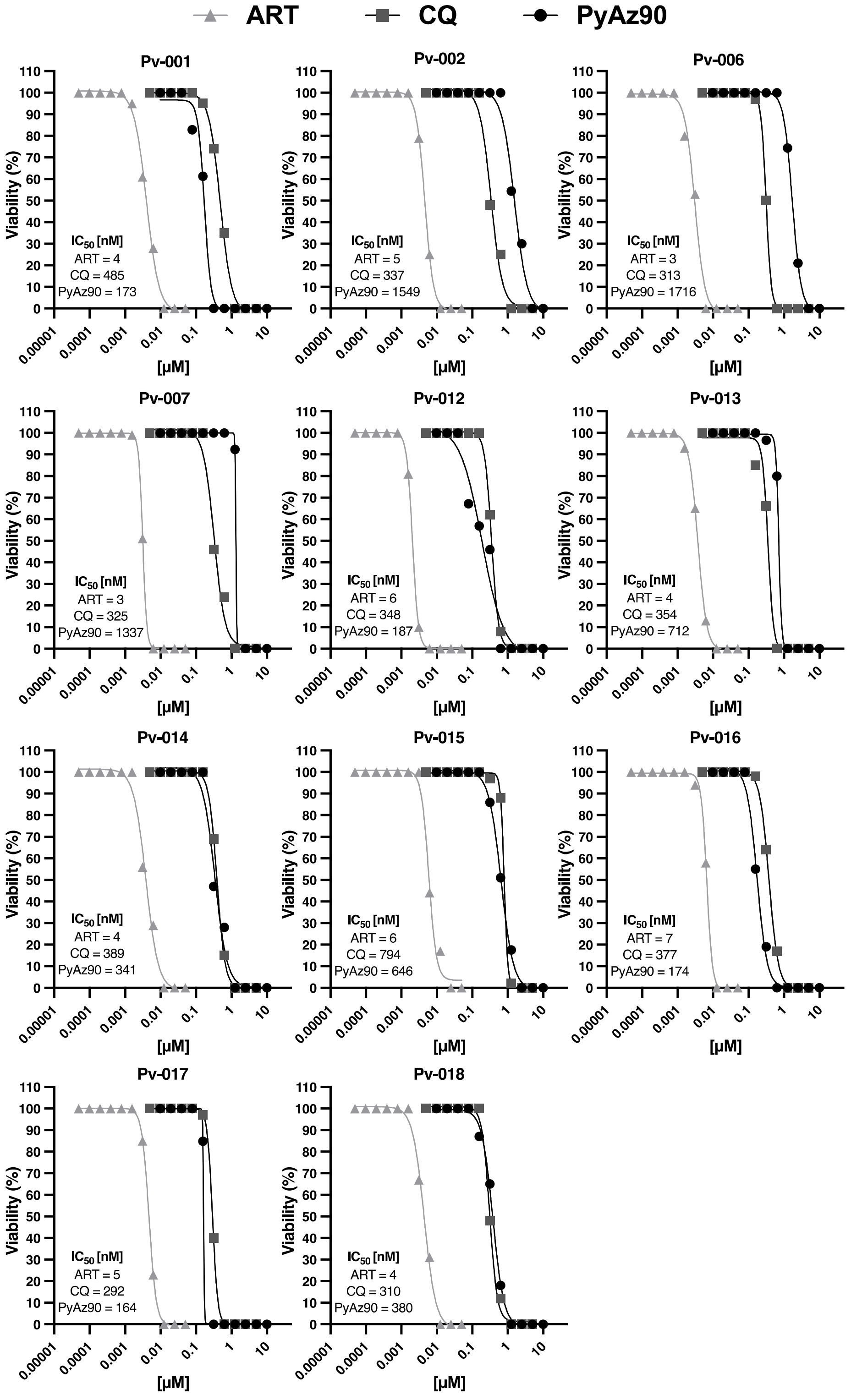

Figure S4: Dose-response curves of PyAz90, chloroquine (CQ) and artesunate (ART) against five *P. falciparum* (Pf) isolates. The parasite viability is normalized to the results from the control wells (DMSO dilutions). IC_{50} values are shown.

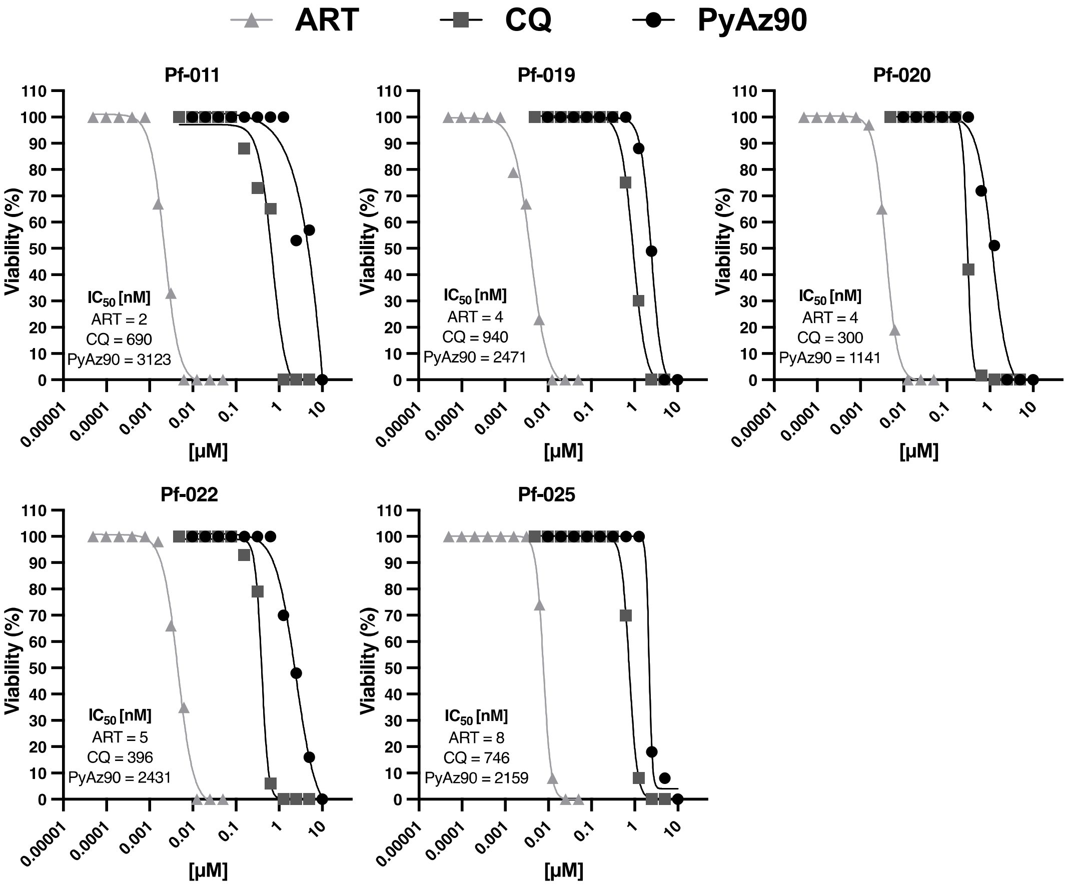

Figure S5: Concentration-response curves and EC_{50} of PyAz90 against asexual stages of the resistant lines generated. Parasites were submitted to 72 h incubation with **PyAz90** in different concentrations in triplicates. The parasite viability is the mean + SD of triplicates for each point normalized to the results from the control wells (DMSO dilutions). The calculated EC_{50} of **PyAz90** for each resistant line and the Dd2_R539T parental, control line is shown.

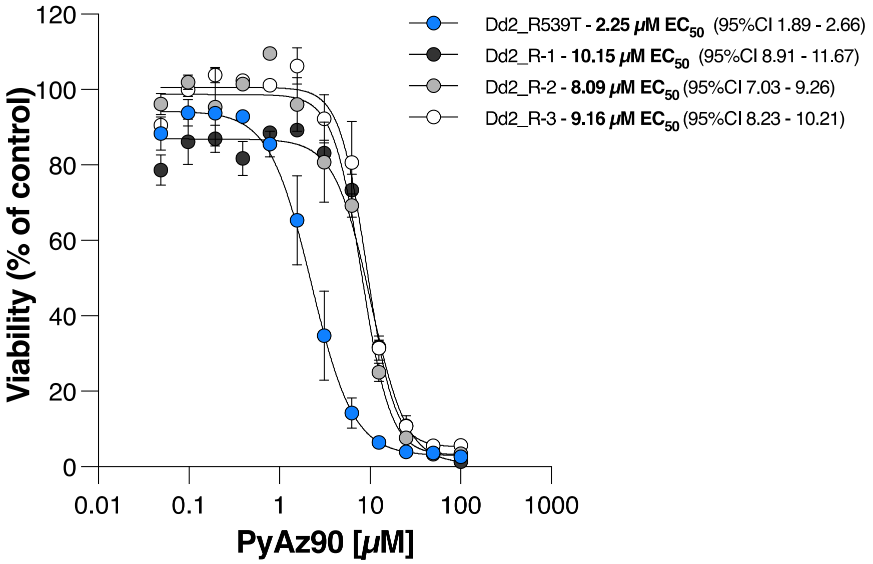

Figure S6: Dose-response curves of PyAz90 and a panel of the indicated antimalarials against *P. falciparum* Dd2_R539T and the PyAz90-resistant Dd2_R1. The parasite viability is the mean of duplicates for each point normalized to the results from the control wells (DMSO dilutions). EC_{50}s values and 95% Confidence Intervals (CI) are shown.

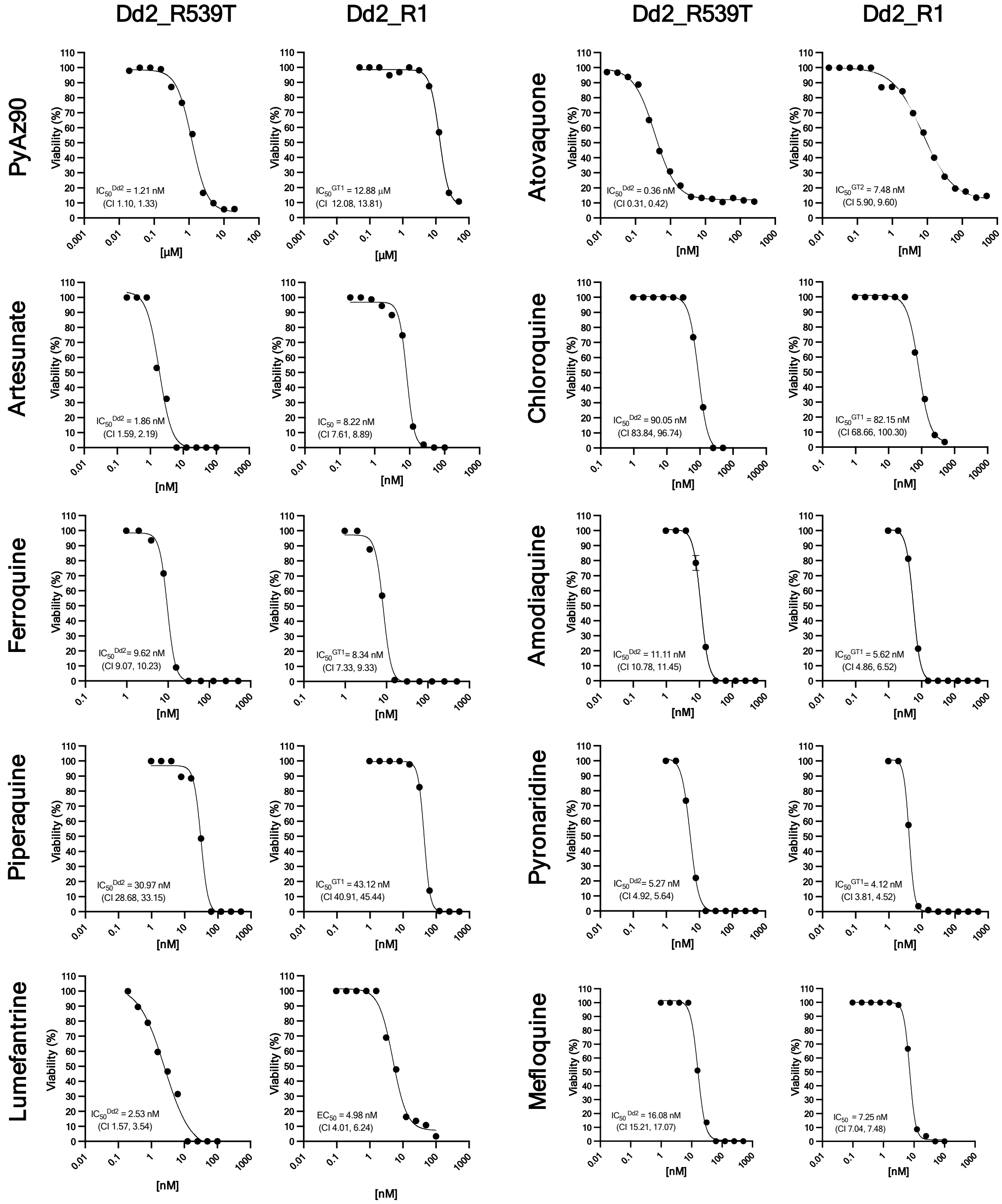

Figure S7. Schematic illustration of **PyAz90** in complex with *Pf*Cyt*b* (Uniprot ID: Q02768), predicted by molecular docking. The residues in orange are Valine 259 and Tyrosine 268, mutated in **PyAz90-** and atovaquone-resistant parasites, respectively. For the ligand: carbon atoms are in gray, nitrogen in blue, oxygen in red, chlorine in green, and iron in cyan.

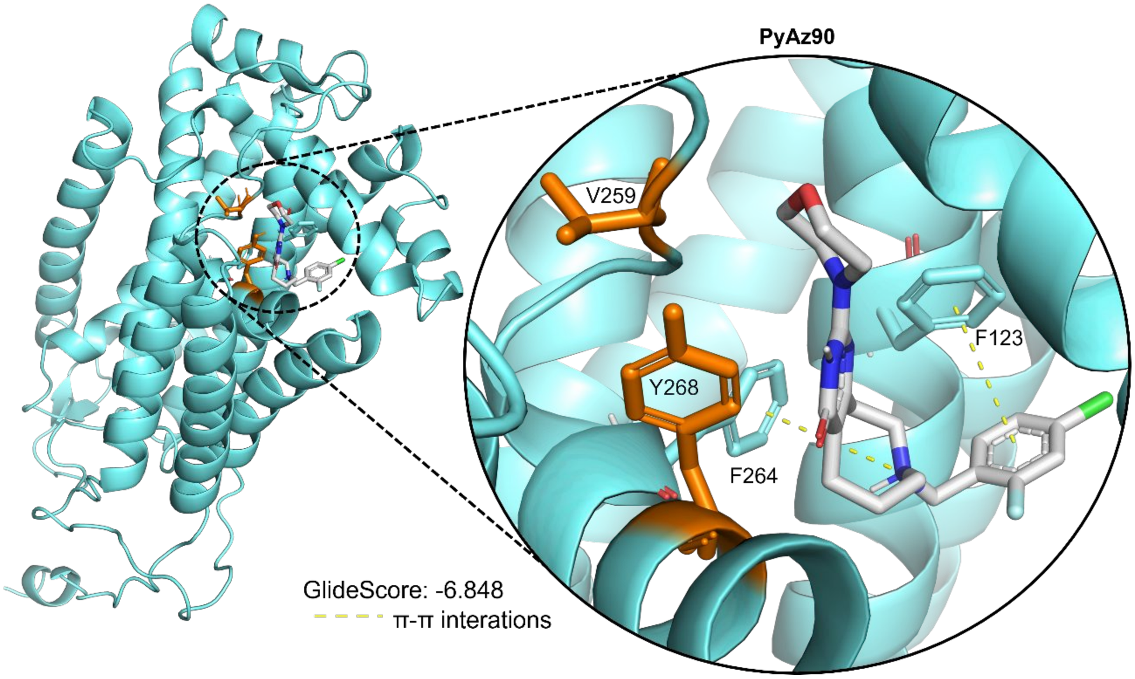

For molecular docking calculations, the **PyAz90** structure was drawn using ChemDraw software v.20.1.1, imported into Maestro workspace v.12.8 (Schrödinger, LCC, New York, 2021), and prepared using the LigPrep tool (LigPrep, Schrödinger, LLC, New York, NY, 2015). All ionization and tautomeric states were generated at pH 7.4 ± 0.5 using Epik software v.5.6 [2, 3] (Schrödinger, LCC, New York, 2021). The lowest potential energy conformers and tautomers were calculated using OPLS4 [4] and retained as input for docking studies.

The predicted 3D structure of *Plasmodium falciparum* cytochrome *b* was obtained from AlphaFoldDB [5, 6], based on the Uniprot ID: Q02768. The generated protein's 3D structure was imported into the Maestro workspace and prepared using the Protein Preparation Wizard tool (Schrödinger, LCC, New York, 2021). Hydrogen atoms were added according to Epik v.5.6, pKa was calculated (pH 7.4 ± 0.5) using PROPKA, and energy minimization was performed using the OPLS4 force field. To generate the grid box, the predicted structure was aligned with the yeast cytochrome *bc*1 complex (PDB ID: 4PD4), the only structure complexed with atovaquone (ATQ) with 3.04 Å of resolution [7]. Then, the outer grid box of 26.98 Å and an inner box of 10 Å around the Q_0 binding site, with the coordinates *x, y* and *z* of 36.75, -30.94, 24.14, respectively, were generated using the receptor grid generation module of the Glide v. 9.1 (Schrödinger, LCC, New York, 2021) available on Maestro workspace (Schrödinger, LCC, New York, 2021). The docking was performed using Glide [8] software v.9.1 on module extra-precision (XP) to generate ten poses for each ligand and the Docking XP score [9]. Finally, to analyze the protein−ligand interactions of the docking poses, we used a PLIP server [10], and to generate the figures, we used Pymol software v. 2.3.0 (www.pymol.org/pymol).

(1) Calit, J.; Araujo, J. E.; Deng, B.; Miura, K.; Gaitan, X. A.; Araujo, M. D. S.; Medeiros, J. F.; Long, C. A.; Simeonov, A.; Eastman, R. T.; et al. Novel Transmission-Blocking Antimalarials Identified by High-Throughput Screening of Plasmodium berghei Ookluc. *Antimicrob Agents Chemother* **2023**, *67* (4), e0146522. DOI: 10.1128/aac.01465-22 From NLM Medline.

(2) Greenwood, J. R.; Calkins, D.; Sullivan, A. P.; Shelley, J. C. Towards the comprehensive, rapid, and accurate prediction of the favorable tautomeric states of drug-like molecules in aqueous solution. *J Comput Aided Mol Des* **2010**, *24* (6-7), 591-604. DOI: 10.1007/s10822-010-9349-1 From NLM Medline.

(3) Shelley, J. C.; Cholleti, A.; Frye, L. L.; Greenwood, J. R.; Timlin, M. R.; Uchimaya, M. Epik: a software program for pK(a) prediction and protonation state generation for drug-like molecules. *J Comput Aided Mol Des* **2007**, *21* (12), 681-691. DOI: 10.1007/s10822-007-9133-z From NLM Medline.

(4) Lu, C.; Wu, C.; Ghoreishi, D.; Chen, W.; Wang, L.; Damm, W.; Ross, G. A.; Dahlgren, M. K.; Russell, E.; Von Bargen, C. D.; et al. OPLS4: Improving Force Field Accuracy on Challenging Regimes of Chemical Space. *J Chem Theory Comput* **2021**, *17* (7), 4291-4300. DOI: 10.1021/acs.jctc.1c00302 From NLM PubMed-not-MEDLINE.

(5) Varadi, M.; Anyango, S.; Deshpande, M.; Nair, S.; Natassia, C.; Yordanova, G.; Yuan, D.; Stroe, O.; Wood, G.; Laydon, A.; et al. AlphaFold Protein Structure Database: massively expanding the structural coverage of protein-sequence space with high-accuracy models. *Nucleic Acids Res* **2022**, *50* (D1), D439-D444. DOI: 10.1093/nar/gkab1061 From NLM Medline.

(6) Varadi, M.; Bertoni, D.; Magana, P.; Paramval, U.; Pidruchna, I.; Radhakrishnan, M.; Tsenkov, M.; Nair, S.; Mirdita, M.; Yeo, J.; et al. AlphaFold Protein Structure Database in 2024: providing structure coverage for over 214 million protein sequences. *Nucleic Acids Res* **2024**, *52* (D1), D368-D375. DOI: 10.1093/nar/gkad1011 From NLM Medline.

(7) Birth, D.; Kao, W. C.; Hunte, C. Structural analysis of atovaquone-inhibited cytochrome bc1 complex reveals the molecular basis of antimalarial drug action. *Nat Commun* **2014**, *5*, 4029. DOI: 10.1038/ncomms5029 From NLM Medline.

(8) Friesner, R. A.; Banks, J. L.; Murphy, R. B.; Halgren, T. A.; Klicic, J. J.; Mainz, D. T.; Repasky, M. P.; Knoll, E. H.; Shelley, M.; Perry, J. K.; et al. Glide: a new approach for rapid, accurate docking and scoring. 1. Method and assessment of docking accuracy. *J Med Chem* **2004**, *47* (7), 1739-1749. DOI: 10.1021/jm0306430 From NLM Medline.

(9) Friesner, R. A.; Murphy, R. B.; Repasky, M. P.; Frye, L. L.; Greenwood, J. R.; Halgren, T. A.; Sanschagrin, P. C.; Mainz, D. T. Extra precision glide: docking and scoring incorporating a model of hydrophobic enclosure for protein-ligand complexes. *J Med Chem* **2006**, *49* (21), 6177-6196. DOI: 10.1021/jm051256o From NLM Medline.

(10) Salentin, S.; Schreiber, S.; Haupt, V. J.; Adasme, M. F.; Schroeder, M. PLIP: fully automated protein-ligand interaction profiler. *Nucleic Acids Res* **2015**, *43* (W1), W443-447. DOI: 10.1093/nar/gkv315 From NLM Medline.
